# Spectral-Based SPD Matrix Representation for Signal Detection Using a Deep Neutral Network

**DOI:** 10.3390/e22050585

**Published:** 2020-05-22

**Authors:** Jiangyi Wang, Xiaoqiang Hua, Xinwu Zeng

**Affiliations:** School of Meteorology and Oceanography, National University of Defence Technology, Changsha 410073, China; wjy05460731@163.com (J.W.); xinwuzeng@nudt.edu.cn (X.Z.)

**Keywords:** signal detection, SPD matrix construction, SPD matrix learning, neural networks

## Abstract

The symmetric positive definite (SPD) matrix has attracted much attention in classification problems because of its remarkable performance, which is due to the underlying structure of the Riemannian manifold with non-negative curvature as well as the use of non-linear geometric metrics, which have a stronger ability to distinguish SPD matrices and reduce information loss compared to the Euclidean metric. In this paper, we propose a spectral-based SPD matrix signal detection method with deep learning that uses time-frequency spectra to construct SPD matrices and then exploits a deep SPD matrix learning network to detect the target signal. Using this approach, the signal detection problem is transformed into a binary classification problem on a manifold to judge whether the input sample has target signal or not. Two matrix models are applied, namely, an SPD matrix based on spectral covariance and an SPD matrix based on spectral transformation. A simulated-signal dataset and a semi-physical simulated-signal dataset are used to demonstrate that the spectral-based SPD matrix signal detection method with deep learning has a gain of 1.7–3.3 dB under appropriate conditions. The results show that our proposed method achieves better detection performances than its state-of-the-art spectral counterparts that use convolutional neural networks.

## 1. Introduction

The essence of signal detection is a binary classification problem to judge whether an input sample contains a target signal. It is the main content of signal detection to find the characteristics of the target signal and distinguish the input sample as only containing interference or existing target signal. Feature-based signal detection is a hotspot in the signal processing field. There are many classic approaches that have helped drive the field, but their shortcomings are also obvious. The instantaneous method [1], which utilizes the instantaneous frequency and amplitude of the signal as the detection target, works on a simple principle but is adversely affected by signal-to-noise ratio or signal-to-clutter ratio (SNR/SCR). The zero-crossing discrimination method [2] has excellent performance at high SNR/SCR levels but requires a high data sampling rate. The maximum likelihood criterion method [3] can theoretically reach optimal performance under the premise of following the Bayesian criterion; however, it relies on prior information. The spectral line feature method [4] requires no prior knowledge and has strong robustness, but its scope of action is limited by the signal spectrum characteristics. These methods all exploit one or more signal spectral characteristic, but are still based on detecting Euclidean space. Under low SNR or SCR conditions, insufficient data, lack of prior information and so on, the detection performances of these methods are still unsatisfactory.

In recent years, the manifold of SPD (symmetric positive definite) matrices has attracted much attention due to its powerful statistical representations. In medical imaging, such manifolds are used in diffusion tensor magnetic resonance imaging [5], and in the computer vision community, they are widely used in face recognition [6,7], object classification [8], transfer learning [9], action recognition in videos [10,11], radar signal processing [12,13,14] and sonar signal processing [15]. The powerful statistical representations of SPD matrices lie in the fact that they inherently belong to the curved Riemannian manifold. Hence, failing to consider the underlying geometric information leads to less than ideal results in practice.

**Figure 1:**
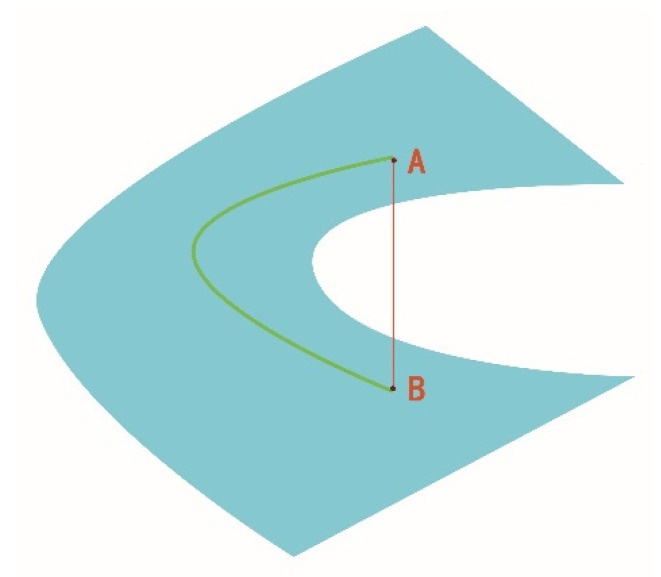
Measurement comparison of the difference between A and B on a manifold of the SPD (symmetric positive definite) matrix.

Figure 1 shows the metric difference between two points on the SPD matrix. The blue surface represents the SPD matrix manifold, and A and B represent two SPD matrices. The red segment represents the Euclidean distance between two points, while the Riemannian distance between two points is represented by the green segment. Euclidean distance measures the nonlinear distance between two points, while the Riemannian distance measures the distance between two points along the matrix manifold, that is, the geodesic distance.

To make full use of the Riemannian geometry characteristics of SPD matrix manifolds, a series of studies have been carried out in the fields of nonlinear metric and matrix learning. To address the problem that SPD matrix manifolds are not applicable to the Euclidean metric, many non-Euclidean metrics are introduced, such as the affine-invariant metric [5] and the log-Euclidean metric [16,17]. Utilizing these Riemannian metrics, a series of methods for working with the underlying Riemannian manifolds have been proposed, for example, flattening SPD matrix manifolds by tangent space approximation [18], mapping SPD matrix manifolds into reproducing kernel Hilbert spaces [19], and pursuing a mapping from an original SPD matrix manifold to another SPD matrix manifold with the same SPD matrix structure to preserve the original SPD matrix geometry [20].

Regarding deep learning, ref. [20] proposed a Riemannian network for SPD matrix learning (SPDnet) that combined a neural network, the method for manifold mapping under the same SPD structure and the log-Euclidean metric. This approach proved to be superior to the traditional neural network method and the shallow SPD matrix learning method. Ref. [21] proposed a deep learning manifold to learn SPD matrices and applied it to face recognition. Ref. [22] proposed some functional improvements on the deep SPD matrix learning network and applied the network to action recognition. In ref. [11], SPDnet was shown to be robust and achieve high accuracy even when the training data are insufficient. Based on deep SPD matrix learning and convolutional neural networks, ref. [23] proposed the SPD aggregation network. It is foreseeable that the combination of deep learning and Riemannian manifolds will be an important development direction of matrix learning.

In this work, we propose a spectral-based SPD matrix signal detection method based on a deep neural network. This method utilizes an SPD matrix deep learning network and two matrix models: an SPD matrix of spectral covariance and an SPD matrix of spectral transformation. This method transforms the problem of signal detection into a binary classification problem on a nonlinear spatial matrix manifold. When nonlinear metrics are applied to signal detection, the differences between the SPD matrices can be found well. Under low SCR conditions, our method outperforms the spectral signal detection method based on a convolutional neural network.

The remainder of this paper is organized as follows: In Section 2, we illustrate that the theory of convolutional neural network is based on Euclidean space and describe the spectral signal detection method based on a convolutional neural network and focus on two classical convolutional neural networks. In Section 3, we illustrate the reasons why the Euclidean metric cannot be used for the SPD matrix and introduce the spectrum SPD matrix signal detection method based on a deep neural network. In Section 4, we first adopt a simulation dataset based on K-distribution clutter to evaluate the performance of the proposed spectral-based SPD matrix signal detection method based on a deep neural network and explore the influences of its hyperparameters, and then adopt a real sea clutter based semi-physical simulation dataset to compare the performance of the signal detection method based on the deep neural network. Finally, a conclusion is provided in Section 5.

## 2. Spectral Image-Based Signal Detection with a Deep Neural Network

The signal detection problem can be transformed into a spectral dichotomy problem, making the methods applicable to image classification also applicable to signal detection. The spectrogram saved in the form of 2-D image is a typical Euclidean structure data [24]. The strong feature extraction abilities of convolutional neural networks give them advantages in image classification and recognition tasks. There is a statistical correlation between the pixel nodes in the image data, and the convolution operation can extract and strengthen the correlation between global nodes or local nodes. Due to the neat arrangement of Euclidean structural data, the different between different samples can be measured by Euclidean metric. The n-dimensional Euclidean space distance is as follows:

(1) $$d\left(x,y\right)=\sqrt{{\left(x_{1}-y_{1}\right)}^{2}+{\left(x_{2}-y_{2}\right)}^{2}+\cdots +{\left(x_{n}-y_{n}\right)}^{2}}=\sqrt{\sum_{i=1}^{n}{\left(x_{i}-y_{i}\right)}^{2}},$$

where d(x,y) denotes the Euclidean distance between two n-dimensional points $X\left(x_{1},x_{2},\ldots ,x_{n}\right)$ and $Y\left(y_{1},y_{2},\ldots ,y_{n}\right)$.

For an image sample with N pixel points, after it is expanded into a vector, it can be regarded as a point in the N-dimensional Euclidean space, and each original pixel point can be regarded as a dimension value of that point. At this point, the difference between different samples is transformed into the distance of different points in N-dimensional Euclidean space. The idea of full connection layer algorithm in deep learning is based on it.

Based on the above characteristics, the convolutional neural network is well established. Since AlexNet [25] first appeared, researchers have developed a series of convolutional neural networks, among which GoogLeNet [26] and ResNet [27] are classic models.

### 2.1. GoogLeNet

GoogLeNet is a type of convolutional neural network proposed by Google in 2014 that won first place in the ILSVRC2014 competition. GoogLeNet has an improved sparse network design, called Inception. Inception has steadily been improved across multiple versions (V1, V2, V3 and V4). Here, we introduce the Inception V1 version used by GoogLeNet.

**Figure 2:**
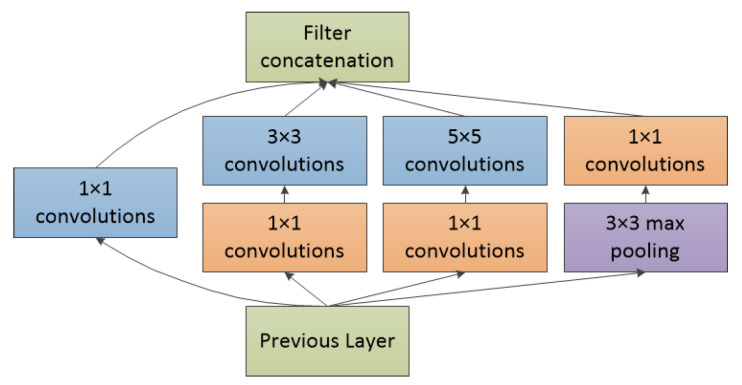
Inception V1 in GoogLeNet.

The Inception module of GoogLeNet originates from a simplified model of the neurons in the human brain. Its structure is shown in Figure 2. Three convolutional layers (blue blocks) are used to perform feature extraction from the input image, and one pooling layer (purple blocks) is used to reduce overfitting. Each convolutional layer is followed by a nonlinear operation that increases the nonlinear characteristics of the network. A 1 × 1 convolutional layer (orange blocks) is used to reduce the feature map dimensions and decrease the number of parameters. The outputs of all the operations are eventually concatenated. GoogLeNet includes nine Inception structures. During training, GoogLeNet uses two auxiliary classifiers to avoid the vanishing gradient problem.

### 2.2. ResNet

ResNet was proposed in 2015 and won first place at ILSVRC2015. Its innovation was the inclusion of a residual block structure that solves the vanishing gradient problem that occurs as the number of neural network layers increases.

ResNet networks currently have evolved through five versions: ResNet18, ResNet34, ResNet50, ResNet101 and ResNet152. In this study, we only use ResNet50.

**Figure 3:**
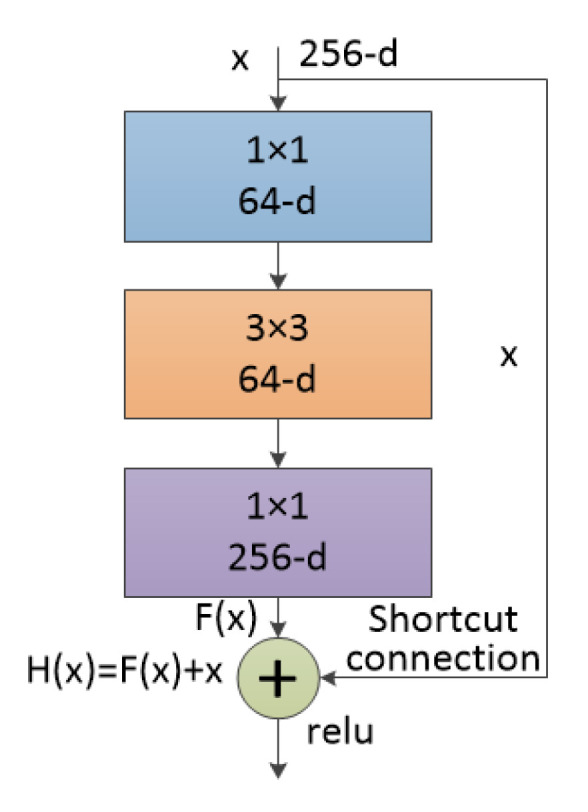
A ResNet50/101/152 residual block.

The residual block structure of ResNet50 is shown in Figure 3, where x is the input of the previous layer. After entering the residual block, x is processed using two methods. The first method conducts a convolution operation, whose output is F(x). The second method takes the input as the output via a shortcut connection. The output of the residual block is H(x)≔F(x)+x. The bulk of ResNet is made up of many residual blocks. The goal when training the network is to make the residual F(x)≔H(x)−x close to 0. Under this training principle, this deep network will not suffer a decline in accuracy, which helps it extract the target characteristics.

The innovations introduced by GoogLeNet and ResNet improved the connection mode of each neural network layer, providing a strong inhibitory effect on the learning degradation caused by deepening the network.

## 3. Spectral-Based SPD Matrix for Signal Detection with a Deep Neural Network

Just as the statistical distribution of image data follows the principle of Euclidean space, the statistical distribution of SPD matrices also follows the principle of Riemannian geometry. The problem of signal detection based on SPD matrix manifold can also be transformed into a dichotomy problem about SPD matrices, but solving the problem of Riemannian geometry by utilizing Euclidean metric will lead to unsatisfactory results. In Section 1, we mentioned several nonlinear metrics respecting Riemannian geometry. They are superior to linear metrics based on Euclidean space in dealing with the problem of SPD matrix manifold. The log-Euclidean metric is widely exploited because of its fast operation speed and stable performance. The Riemannian nonlinear metric used in this paper is a log-Euclidean metric. We exploit two methods to construct SPD matrices, which we call them spectrum transformation and spectrum covariance, respectively.

### 3.1. Spectral-Based SPD Matrix Construction

#### 3.1.1. SPD Matrix Construction Method Based on Spectrum Transformation

The following eigen-decomposition can be carried out on a square matrix A:

(2) $$A=U\Sigma U^{-1},$$

where Σ is the eigenvalue diagonal matrix of A, and U is a full matrix whose columns are the corresponding eigenvectors.

The eigenvalues and eigenvectors of the square matrix reflect the matrix properties. Eigenvalue decomposition is not applicable to a non-square matrix B; instead, it is replaced by singular value decomposition:

(3) $$B=U\Sigma V^{T},$$

where Σ is the singular value diagonal matrix of B, U is a full matrix whose columns are the corresponding eigenvectors of $BB^{T}$, and V is a full matrix whose columns are the corresponding eigenvectors of $B^{T}B$.

Then, the non-Euclidean properties of the SPD matrix manifold are used to classify the set of B with different information. This method also applies to square matrices.

Based on the above ideas, we propose the threshold method for constructing an SPD matrix:

(4) $$X_{1}=X_{0}X_{0}^{T}\left(or\;X_{1}=X_{0}^{T}X_{0}\right)$$

(5) $$X_{1}=U_{1}\Sigma_{1}U_{1}^{-1}$$

(6) $$X_{2}=U_{1}\max\left(\varepsilon I,\Sigma_{1}\right)U_{1}^{-1},$$

where $U_{1}$ and $\Sigma_{1}$ are obtained by eigenvalue decomposition, $X_{1}=U_{1}\Sigma_{1}U_{1}^{-1}$, $X_{0}$ is the sample image, and ε is a rectification threshold (in this paper, ε is set to $1\times 10^{-5})$.

The steps to transform the spectrum into the SPD matrix are as follows: first, the original time domain sequence is transformed into the energy distribution based on time axis and frequency axis in the form of two-dimensional matrix using the short-time Fourier transform, and the modulus of such matrix is retained to generate sample $X_{0}$. Then, each sample $X_{0}$ is calculated by Formula (4) to generate SPD matrix $X_{2}$.

#### 3.1.2. SPD Matrix Construction Method Based on Spectrum Covariance

The authors of [28] proposed the following method for constructing an SPD matrix set based on the covariance matrix:

(7) $$C=\frac{1}{n-1}\sum_{i=1}^{n}\left(s_{i}-\bar{s}\right){\left(s_{i}-\bar{s}\right)}^{T},$$

where $s_{i}$ denotes the i-th image sample with d-dimensional feature description in the data matrix of an image set with n samples, $S=\left[s_{1},s_{2},\ldots ,s_{n}\right]$, and $\bar{s}$ is the mean of the image samples.

The covariance matrix constructed by this method follows the properties of the SPD matrix. Inspired by it, we propose the SPD matrix construction method for spectral transformation, which is called spectral covariance in this paper. The steps of this method are as follows:

(8) $$Y_{1}=\frac{1}{n-1}\sum_{i=1}^{n}\left(X_{i}-\bar{X}\right){\left(X_{i}-\bar{X}\right)}^{T},$$

(9) $$Y_{2}=U_{1}\max\left(\varepsilon I,\Sigma_{1}\right)U_{1}^{-1},$$

where $X_{i}$ denotes the i-th spectrum sample in the spectrum group with n samples, $X=\left[X_{1},X_{2},\ldots ,X_{n}\right]$, and $\bar{X}$ is the mean of the spectrum samples in this group, $U_{1}$ and $\Sigma_{1}$ are obtained by eigenvalue decomposition, $Y_{1}=U_{1}\Sigma_{1}U_{1}^{-1}$, and ε is a rectification threshold. In this paper, ε is set to $1\times 10^{-5}$ and n is set to 8.

The steps to transform the spectrum into the SPD matrix are as follows: first, the original time-domain sequence is transformed into a time-frequency joint energy distribution in the form of a two-dimensional matrix by short-time Fourier transform, and their modulus matrix is retained. Then, the modulus matrix generated in the previous step is transformed into $Y_{1}$ by using Formula (6). In order to ensure positive characterization, Formula (7) is used to retain the appropriate matrix eigenvalues.

The covariance method has some disadvantages. First, it describes the correlations between samples; however, the use of correlation means that constructing an SPD matrix requires multiple raw samples. Thus, this constructor is not suitable for addressing situations with insufficient data. Second, averaging calculation performed in the covariance method may result in the loss of some features. These deficiencies reduce the performance of the SPD matrix learning method based on a deep neural network.

### 3.2. SPDnet

Similar to classical convolutional neural networks, SPDnet also designs convolutional-liked layers and rectified linear units-like layers, named bilinear mapping (BiMap) layers and eigenvalue rectification (ReEig) layers, respectively. BiMap layers aim to make the input SPD matrix manifolds more compact and more discriminative and ReEig layers aim to rectify the upper input SPD matrices with a non-linear function. After BiMap layers and ReEig layers, the input SPD matrices will be converted into SPD matrices that follow a more compact and discriminative Riemannian manifold distribution. The formula for a BiMap layer is as follows:

(10) $$X_{k}=W_{k}X_{k-1}W_{k}^{T},$$

where $X_{k-1}\in Sym_{d_{k-1}}^{+}$ is the input matrix of the k-th layer, $W_{k}\in {\mathbb{R}}_{*}^{+},(d_{k}<d_{k-1})$ is a weight matrix, which is a row full-rank matrix, and $X_{k}\in Sym_{d_{k}}^{+}$ is the resulting matrix.

The formula for a ReEig Layer is

(11) $$X_{k}=U_{k-1}\max\left(\varepsilon I,\Sigma_{k-1}\right)U_{k-1}^{T},$$

where $U_{k-1}$ and $\Sigma_{k-1}$ are achieved by eigenvalue decomposition, $X_{k-1}=U_{k-1}\Sigma_{k-1}U_{k-1}^{T}$, ε is a rectification threshold, I is an identity matrix, and $\max\left(\varepsilon I,\Sigma_{k-1}\right)$ means preserving the elements in $\Sigma_{k-1}$ that are greater than ε and replacing the rest with ε.

The LogEig layer is exploited to perform Riemannian computing on the resulting SPD matrices. It utilizes the log-Euclidean Riemannian metric to reduce the Riemannian manifold of SPD matrices to a flat space with the matrix logarithm operation log(·) on the SPD matrices. After the LogEig layer, the metric difference of SPD matrices in Riemannian manifold can be reflected in Euclidean space. The linear classification method based on Euclidean space can be used. The formula for the LogEig layer is

(12) $$X_{k}=U_{k-1}log\left(\Sigma_{k-1}\right)U_{k-1}^{T},$$

where $X_{k-1}=U_{k-1}\Sigma_{k-1}U_{k-1}^{T}$ is an eigenvalue decomposition and $\log\left(\sum_{k-1}\right)$ is the diagonal matrix of eigenvalue logarithms.

**Figure 4:**
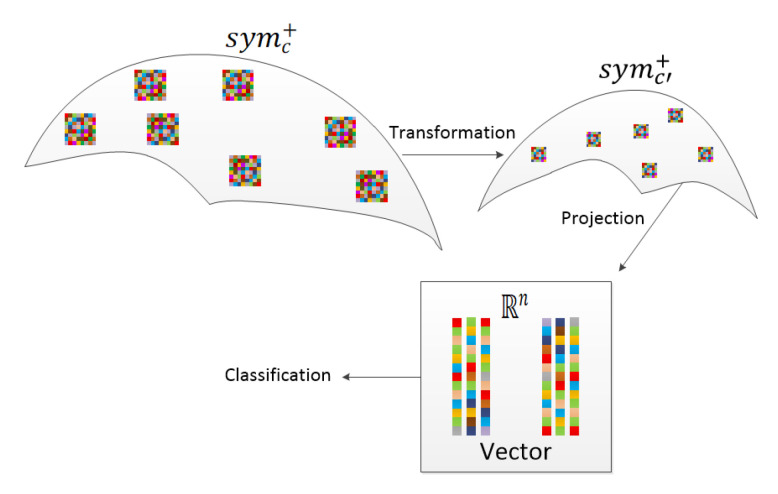
SPDnet workflow.

Figure 4 shows the SPDnet workflow. After several successive bilinear mapping layers and rectified eigenvalue layers, the generated SPD matrices with more compact features will be projected to Euclidean space by the log-Euclidean metric. Eventually, they are classified within Euclidean space. In this paper, we mainly adopt an 8-layer SPDnet model: $X_{0}\to f_{BM}^{1}\to f_{RE}^{2}\to f_{BM}^{3}\to f_{RE}^{4}\to f_{BM}^{5}\to f_{LE}^{6}\to f_{FC}^{7}\to f_{soft}^{8}$, where $X_{0},f_{BM},f_{RE},f_{LE},f_{FC},f_{soft}$ indicate the input SPD matrix, BiMap, ReEig, LogEig, fully-connected and softmax log-loss layers, respectively.

The main difficulties in training SPDnet lie both in backpropagation through the structured Riemannian functions and in manifold-constrained optimization. For more details, please refer to [12,21].

## 4. Results

### 4.1. Experimental Analysis of Simulation Data

In the framework of a deep neural network, we compared three signal detection methods: the signal detection method based on time-frequency spectrum images, the SPD matrix signal detection method based on spectral covariance and the SPD matrix signal detection method based on spectral transformation. The data were derived from a simulation signal dataset with K-distribution clutter as interference.

K-distribution is the most recognized model of real sea clutter radiation. It takes the correlation between pulses into account, well fits the amplitude distribution of sea clutter, and well explains the scattering mechanism of sea clutter. K-distribution model is composed of the texture component with slow change following the Gamma distribution and the speckle component with fast change following the Rayleigh distribution. The probability density function of K-distribution is

(13) $$f\left(x\right)=\frac{2}{a\Gamma \left(v\right)}{\left(\frac{x}{2a}\right)}^{v}K_{v-1}\left(\frac{x}{a}\right),$$

where $K_{v-1}\left(\cdot \right)$ is the second modified Bessel function of order v−1, Γ(·) is the Gamma function, a is the scale parameter, representing the mean value of clutter, v is the shape parameter, affecting the shape of the distribution curve. In this paper, a is set to 1 and v is set to 1.

We use a simulated target signal in our experiment. The driving vector of the target signal is

(14) $$p=\frac{1}{\sqrt{N}}{\left[1,\exp\left(j2\pi f_{d}\right),\ldots ,\exp\left(j2\pi \left(N-1\right)f_{d}\right)\right]}^{T},$$

where N is the number of impulses of target signal, $f_{d}$ is the normalized Doppler frequency. In this paper, N is set to 2048, and $f_{d}$ is set to 0.15.

The normalized doppler frequency is defined as follows:

(15) $$f_{d}=\frac{F_{d}}{f_{s}}=\frac{vf_{c}}{cf_{s}},$$

where $F_{d}=\frac{vf_{c}}{c}$ is the doppler frequency of the target, v is the speed of the target relative to the receiving end, c is the speed of the radar, $f_{c}$ is the carrier frequency of the radar, and $f_{s}$ is the pulse repetition rate of the radar. In this paper, v is set to 5 m/s, c is set to, $f_{c}$ is set to 9.39 GHz, and $f_{s}$ is set to 1000 Hz.

**Figure 5:**
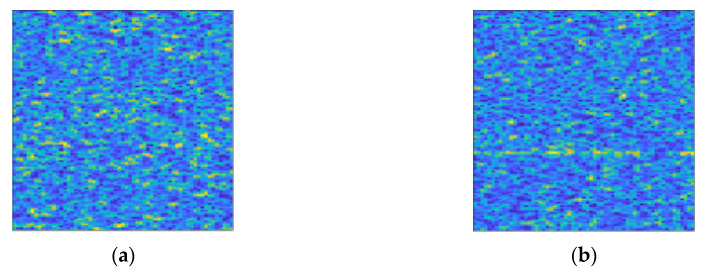
Colour maps of the time-frequency spectrum used in this study. (**a**) The time-frequency spectrum colour map of clutter; (**b**) The colour map of the time-frequency spectrum of target signal interfered by clutter. The SCR in it is −13 dB. And the trace of the target signal can be found in the lower part of the figure, but it is blurred by clutter.

The dataset is divided into two categories: clutter only and signal interfered by clutter. The clutter only consists of 11,000 samples. The signal interfered by clutter consists of a series of samples with different SCR distribution ranges from −5 dB to −30 dB at intervals of 1 dB, each with 5000 samples. Figure 5 shows the colour map of the time-frequency spectrum used in this study.

#### 4.1.1. Comparison with Convolutional Neural Networks

**Figure 6:**
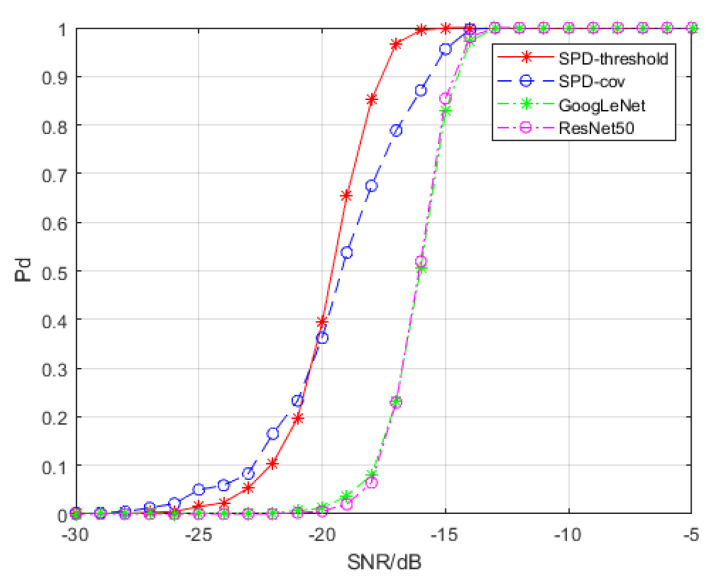
Detection probability curves of different models. The false alarm probability of all the models is controlled to be less than $10^{-4}$. “SPD-threshold” represents the spectral transformation SPD matrix network, and “SPD-cov” represents the spectral covariance SPD matrix network.

We exploit GoogLeNet and ResNet50 to detect the target signal in time-frequency spectra and SPDnet to detect the target signal in SPD matrices. For GoogLeNet and ResNet50, we used transfer learning to adjust the fully connected layers to fit our detection problem, and SPDnet is retrained. The data used for transfer learning and retraining are clutter samples and signal samples with SCRs of −5, −10, −15, −20, −25 and −30 dB, respectively. Each training set includes 1000 clutter samples and 1000 signal samples with the same SCR. We used 4000 signal samples for calculating detection probability at each SCR level that had no overlap with the samples used for training. The hyperparameters involved in training are as follows: the batch size is 20, the learning rate is 0.001, and the weight decay is 0.0005. For SPDnet we used an epoch of 500; the epochs for GoogLeNet and ResNet50 were 2000. Under these values, the accuracy of the models is guaranteed to reach saturation during the training processes. We trained SPDnet on an i7-8565U CPU, while GoogLeNet and ResNet50 were trained on an Nvidia GTX 960 M GPU. The detection probability results are depicted in Figure 6.

Figure 6 shows that the spectral transformation SPD matrix network achieves a detection probability of 80% at approximately −18.3 dB, while GoogLeNet and ResNet50 under time-frequency spectra as training and testing samples achieve a detection probability of 80% at approximately −15 dB. Thus, we can conclude that the spectral transformation SPD matrix network achieves an improvement of 3.3 dB compared to that of the time-frequency spectra. The spectral covariance SPD matrix network also outperforms the time-frequency spectra, but its performance is inferior to that of the spectral transformation SPD matrix network after −20 dB.

Table 1 compares the variation in the false alarm probability for several deep network models based on different inputs under different SCR levels. They are calculated using 10,000 clutter samples. As the SCR decreases, the growth rate of the false alarm probability of the two SPD matrix detection networks is slower than those of GoogLeNet and ResNet50.

A comparison of the two SPD matrix detection networks showed that when the false alarm probability of the spectral transformation SPD matrix network is greater than $10^{-4}$, its SCR is smaller than that of the spectral covariance SPD matrix network. However, when the false alarm probability of the spectral covariance SPD matrix network is greater than $10^{-4}$, its false alarm probability is greater than that of the spectral covariance SPD matrix network.

Table 2 compares the training time of different models. The total training time is the average sum of each training time. The training time of SPDnet is much less than that of the complex convolutional neural network models.

#### 4.1.2. Comparison with Convolutional Neural Networks

Hyperparameters affect the learning performances of deep networks. In this section, we study the influence of two hyperparameters, the learning rate and the weight attenuation coefficient, on the performance of the spectral-based SPD matrix signal detection method with deep learning and study the relationship between the number of network layers and the spectral-based SPD matrix signal detection method with deep learning. For simplicity, we exploit only the spectral transformation SPD matrix network as the research target. The false alarm probability in all the experimental combinations involved in the detection probability comparison is less than $10^{-4}$.

**Figure 7:**
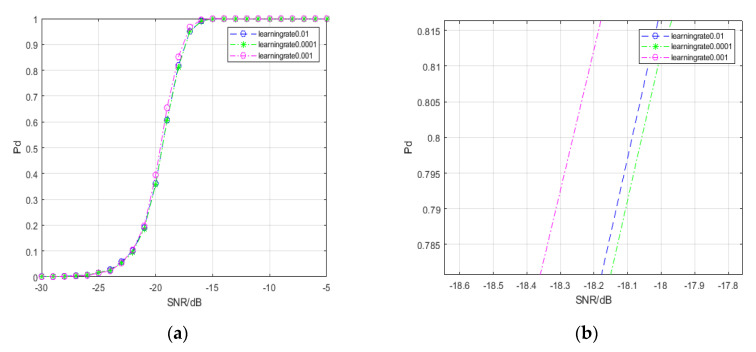
Detection probability curves under different learning rates. (**a**) reveals the general trends of three curves; (**b**) reveals the differences in the three curves when the detection probability is approximately 80%.

Figure 7 shows the detection probability curves of the spectral-based SPD matrix signal detection method under different learning rates. When the SCR ranges from −21 dB to −14 dB, the detection probability at a learning rate of 0.001 is higher than in the other two cases. When the detection probability is 80%, the spectral-based SPD matrix signal detection method with the learning rate set to 0.001 achieves a gain of approximately 0.2 dB. Thus, a learning rate that is too large or too small may not result in ideal performance on the signal detection problem.

**Figure 8:**
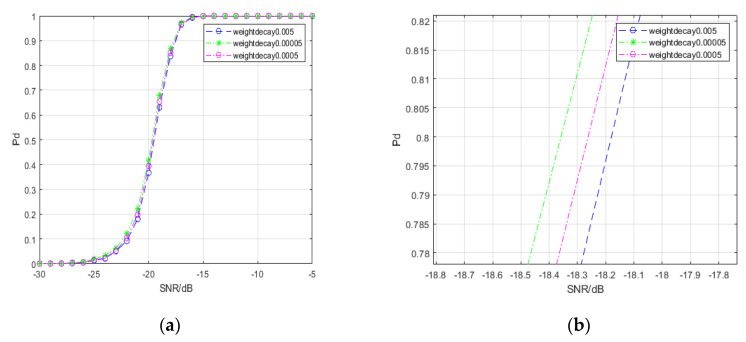
Detection probability curves under different weight decays. (**a**) reveals the general trends of three curves; (**b**) reveals the differences in the three curves when the detection probability is approximately 80%.

Weight decay is a method to prevent overfitting of deep network models. By gradually reducing the weight to a smaller value, the deep network model overfitting problem can be reduced. The overfitting problem occurs when the network’s performance is good in training but poor in testing. In our study, one of the hyperparameters is the magnitude of weight decay. Figure 8 shows the detection probability curves of the spectral-based SPD matrix signal detection method under different weight decay values.

From Figure 8, we find that a smaller weight decay value is conducive to improving the signal detection performance of the spectral-based SPD matrix signal detection method.

**Figure 9:**
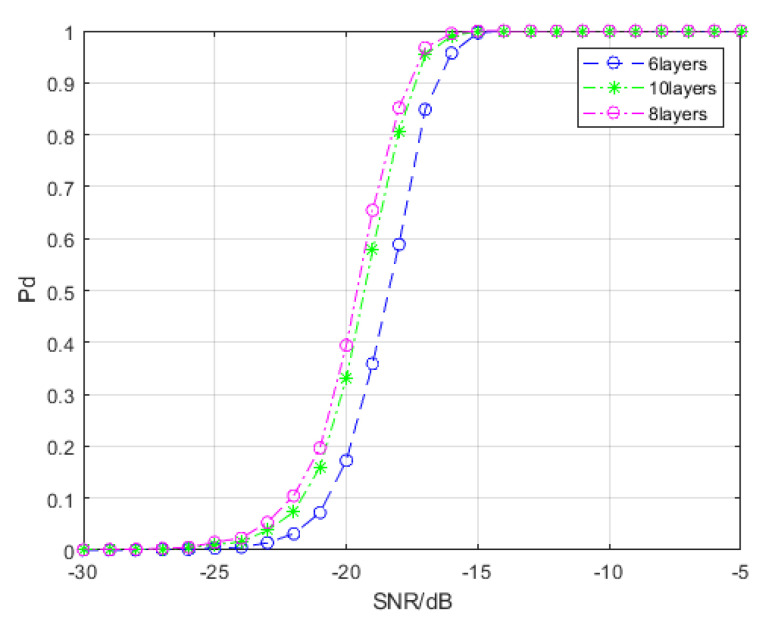
Detection probability curves of the spectral-based SPD matrix signal detection method under different network layers.

For a deep neural network, differences in the number of layers can affect model performance. We conducted a comparative experiment to investigate the ideal number of layers; the results are shown in Figure 9. The 8-layer model is consistent with the model used in Section 4.1. The 10-layer model adds a BiMap layer and a ReEig layer, while the 6-layer model removes a BiMap layer and a ReEig layer. We find that the performance of the 8-layer model is the best; adding or removing specific functional layers is not conducive to improving the signal detection probability. Excessive SPD matrix aggregation operations may result in the loss of information characteristics, while a reduction of the number of layers may reduce the parameters, which is not conducive to sample classification based on the SPD matrix manifold.

Table 3, Table 4 and Table 5 show the false alarm probability of the spectral-based SPD matrix signal detection method under different SCRs when changing different hyperparameters. The results in Table 3 and Table 4 show that learning rate and weight decay have little influence on changing the false alarm probability of the spectral-based SPD matrix signal detection method, while the results in Table 5 show that reducing the number of layers will lead to an unreasonable false alarm probability by the spectral-based SPD matrix signal detection method at a higher SCR. This indicates that the SPD matrix feature aggregation ability and the classification performance of the network model with fewer layers are poor. Note that too many aggregation layers also reduce the network’s ability to discriminate among SPD matrices with weak target signals. This can explain why the false alarm probability of the 10-layer model in Table 5 is higher than that of the 8-layer model at −20, −25 and −30 dB.

By comparing the results of the detection probability curves, when the number of iterations is sufficient, the influence of the learning rate on the detection probability is greater than its influence on the false alarm probability.

### 4.2. Experimental Analysis of Semi-Physical Simulation Data

**Figure 10:**
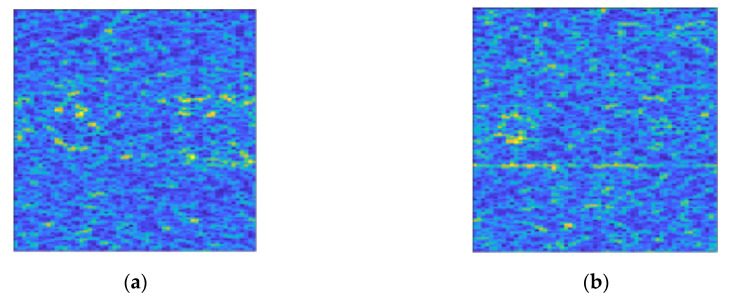
Time-frequency spectrum colour map based on IPIX sea clutter. Different from the clutter based on K-distribution simulation, the distribution of IPIX sea clutter in time and frequency is obviously uneven. (**a**) The time-frequency spectrum colour map of clutter; (**b**) The colour map of the time-frequency spectrum of target signal interfered by clutter. The SCR in it is −13 dB. And the trace of the target signal can be found in the lower part of the figure, but it is blurred by clutter.

To further compare the detection performance of the different models, we utilize the lake IPIX (Ice Multiparameter Imaging X-Band) radar data file collected by McMaster University in Ontario on February 23, 1998 as the clutter [29]. The referenced radar data files and their parameters are shown in Table 6 and Table 7. Since there is no clear target information in the original data, we add a simulation target. The pulse number of each sample is set to 2000, and the remaining parameters are the same as those in Section 4.1. Figure 10 shows the colour map of the time-frequency spectrum used in this section.

Table 8 compares the false alarm probabilities of different models under different SCR. When the time-frequency distribution of sea clutter is not uniform, the false alarm probability of both SPD matrix detection networks increases significantly, especially the detection network based on covariance SPD matrix. The two models of convolutional neural network are superior to the two proposed SPD matrix detection networks in suppressing false alarm.

**Figure 11:**
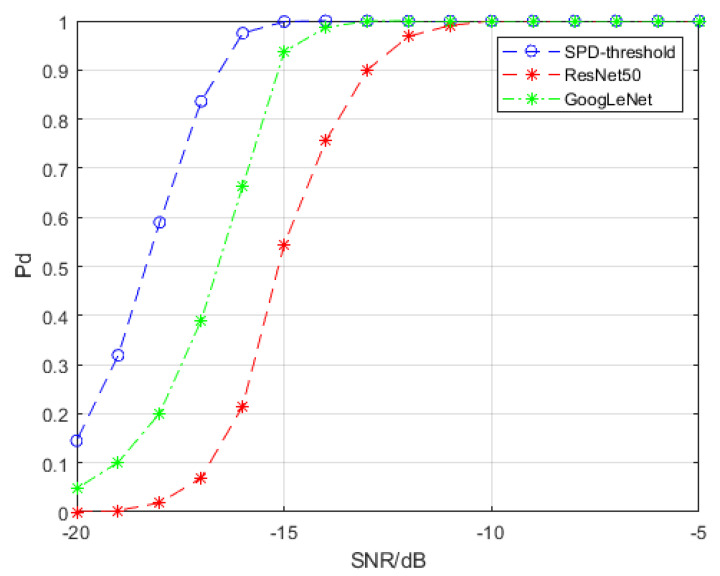
Detection probability curves of different models. The false alarm probability of all the models is controlled to be less than $10^{-4}$. “SPD-threshold” represents the spectral transformation SPD matrix network.

Figure 11 compares the detection performance of SPD matrix detection network with that of convolutional neural network. Due to the high false alarm probability, the covariance SPD matrix network does not participate in this comparison. Compared with Section 4.1, the detection performance of all models decreases, but remain stable. When the detection probability is 70%, the SPD matrix detection network based on spectral transformation has a gain of 1.7 dB compared with GoogLeNet and 3.3 dB compared with ResNet50. According to the false alarm probability of GoogLeNet and ResNet50, the decline of detection performance of these two kinds of convolutional neural networks is due to the serious omission of detection. The non-uniformity of clutter distribution may affect the recognition of signal samples by convolutional neural network.

## 5. Conclusions

In this paper, we proposed a spectral-based SPD matrix signal detection method based on a deep network. Using the SPD matrix of spectral transformation and combined with the deep SPD matrix learning network, the proposed method can detect a target signal even under low SCR. The advantages of this method are that it exploits the nonlinear spatial property of the SPD matrix space as a Riemannian manifold, increases the discriminability of different matrices, and uses a nonlinear metric to achieve the dichotomy. Simultaneously, the adoption of deep learning enhances the method’s ability to aggregate the SPD matrices and adjust the parameters to adapt to signal detection problems. We used a simulation signal dataset based on K-distribution clutter and a semi-physical simulation signal dataset based on real sea clutter to test our proposed method. The results showed that our method outperforms the signal detection method based on a convolutional neural network. And the gains realized based on the simulation dataset and the semi-physical simulation dataset are 3.3 dB and 1.7 dB, respectively. We also studied the performance impacts of the hyperparameters on our method. Because our method is based on time-frequency spectra transformation, it can be applied to both signal processing and image detection tasks.

## Figures and Tables

**Table 1:** False alarm probability curves of different models (expressed as %).

	Model	Spectral Transformation SPD Matrix Network	Spectral Covariance SPD Matrix Network	GoogLeNet with Time-Frequency Spectra	ResNet50 with Time-Frequency Spectra
SCR(dB)					
−5		Less than 0.01	Less than 0.01	Less than 0.01	Less than 0.01
−10		Less than 0.01	Less than 0.01	Less than 0.01	Less than 0.01
−15		Less than 0.01	0.23	1.12	0.50
−20		2.47	2.22	32.97	25.95
−25		18.80	16.65	99.96	39.01
−30		29.80	14.00	99.98	49.15

**Table 2:** Training time of different models.

Model	Spectral Transformation SPD Matrix Network	Spectral Covariance SPD Matrix Network	GoogLeNet with Time-Frequency Spectra	ResNet50 with Time-Frequency Spectra
Total Training Time(Min)	67.6	69.5	2068.0	7083.3
Total Number of Epochs	500	500	2000	2000
The Average Time per 100 Epochs(Min)	13.5	13.9	103.4	354.2

**Table 3:** False alarm probability curves of different learning rates (expressed as %).

	Model	Learning Rate 0.01	Learning Rate 0.001	Learning Rate 0.0001
SCR(dB)				
−5		Less than 0.01	Less than 0.01	Less than 0.01
−10		Less than 0.01	Less than 0.01	Less than 0.01
−15		Less than 0.01	Less than 0.01	Less than 0.01
−20		8.02	2.50	12.42
−25		15.51	19.44	33.00
−30		26.38	29.98	37.00

**Table 4:** False alarm probability curves of different weight decays (expressed as %)

	Model	Weight Decay 0.005	Weight Decay 0.0005	Weight Decay 0.00005
SCR(dB)				
−5		Less than 0.01	Less than 0.01	Less than 0.01
−10		Less than 0.01	Less than 0.01	Less than 0.01
−15		Less than 0.01	Less than 0.01	Less than 0.01
−20		9.94	2.50	9.93
−25		23.36	19.40	23.5
−30		27.28	29.98	27.28

**Table 5:** False alarm probability curves of different layers (expressed as %).

	Model	6 Layers	8 Layers	10 Layers
SCR(dB)				
−5		Less than 0.01	Less than 0.01	Less than 0.01
−10		Less than 0.01	Less than 0.01	Less than 0.01
−15		0.08	Less than 0.01	Less than 0.01
−20		4.82	2.51	6.13
−25		26.33	19.44	24.08
−30		28.10	30.10	28.42

**Table 6:** The IPIX (Ice Multiparameter Imaging X-Band) radar data file used in this section.

Number	Name
1	19980223_171533_ANTSTEP
2	19980223_171811_ANTSTEP
3	19980223_172059_ANTSTEP
4	19980223_172410_ANTSTEP
5	19980223_172650_ANTSTEP
6	19980223_184853_ANTSTEP
7	19980223_185157_ANTSTEP

**Table 7:** Parameters of IPIX sea clutter signal.

Pulse Repetition Frequency	Carrier Frequency	The Length of the Pulse	Range Resolution	Polarization Mode
1000 Hz	9.39 GHz	60,000	3 m	HH

**Table 8:** False alarm probability curves of different models (expressed as %).

	Model	Spectral Transformation SPD Matrix Network	Spectral Covariance SPD Matrix Network	GoogLeNet with Time-Frequency Spectra	ResNet50 with Time-Frequency Spectra
SCR(dB)					
−5		Less than 0.01	0.25	Less than 0.01	Less than 0.01
−10		Less than 0.01	0.42	Less than 0.01	Less than 0.01
−15		0.08	2.17	0.15	Less than 0.01
−20		6.90	13.92	14.49	10.52
